# Terpenes from Marine-Derived Fungi

**DOI:** 10.3390/md8082340

**Published:** 2010-08-13

**Authors:** Rainer Ebel

**Affiliations:** Marine Biodiscovery Centre, University of Aberdeen, Meston Walk, Aberdeen, AB24 3UE, Scotland, UK; E-Mail: r.ebel@abdn.ac.uk; Tel.: +44-1224-27-2930; Fax: +44-1224-27-2921

**Keywords:** marine-derived fungi, natural products chemistry, terpenes, biological activity

## Abstract

Terpenes from marine-derived fungi show a pronounced degree of structural diversity, and due to their interesting biological and pharmacological properties many of them have aroused interest from synthetic chemists and the pharmaceutical industry alike. The aim of this paper is to give an overview of the structural diversity of terpenes from marine-derived fungi, highlighting individual examples of chemical structures and placing them in a context of other terpenes of fungal origin. Wherever possible, information regarding the biological activity is presented.

## 1. Introduction

Marine-derived fungi continue to produce chemically diverse new natural products with interesting pharmacological properties [1–3]. In terms of the overall number of secondary metabolites, polyketides are clearly dominating the chemical literature, followed by prenylated polyketides (“meroterpenes”), peptides and alkaloids. Terpenes, which are in the focus of this special issue of *Marine Drugs*, thus seem to play a less conspicuous role in the natural products chemistry of fungi from marine environments, but as will be shown, this lack in quantity does not translate into a lack in quality.

The aim of this paper is to provide an overview of the structural diversity of terpenes from marine-derived fungi, highlighting individual examples of chemical structures and placing them in a context of other terpenes of fungal origin. Wherever possible, information regarding the biological activity is presented. Without claiming to achieve comprehensive coverage, the focus will exclusively be on true terpenes, divided into the classical biogenetic subclasses, *i.e.*, monoterpenes, sesquiterpenes, diterpenes, sesterterpenes, triterpenes including steroids, and tetraterpenes or carotenoids. Excluded from this are natural products that only in part consist of a terpene-derived skeleton, most importantly prenylated polyketides (“meroterpenes”), but also prenylated alkaloids or prenylated peptides, of which there are numerous examples.

## 2. Examples of Terpenoids

### 2.1. Monoterpenes

Monoterpenes have only rarely been reported from fungi in general, let alone marine-derived strains. A notable exception is the new chlorinated monoterpene, (1*S*,2*S*,3*S*,4*R*)-3-chloro-4-(2- hydroxypropan-2-yl)-1-methylcyclohexane-1,2-diol (**1**) which was isolated from the fermentation broth of the mangrove endophytic fungus *Tryblidiopycnis* sp., obtained from *Kandelia* woody tissue in Hong Kong [4].

**Figure:**
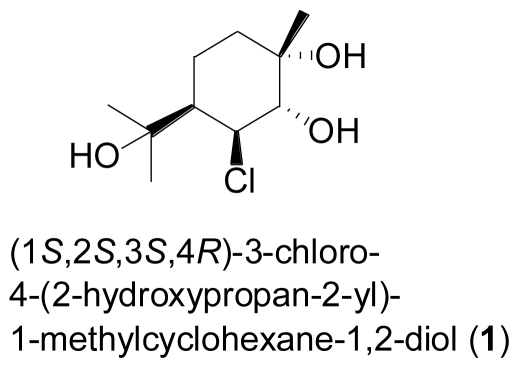


### 2.2. Sesquiterpenes

The first new terpene at all from marine-derived fungi was dendryphiellin A (**2**) from the obligate marine deuteromycete *Dendryphiella salina* [5]. Compound **2** is structurally unusual in two ways— firstly, at the time of its isolation it represented the first trinor-eremophilane identified from fungi so far, and moreover, also its fatty acid-derived ester side chain had not previously been reported from fungi.

**Figure:**
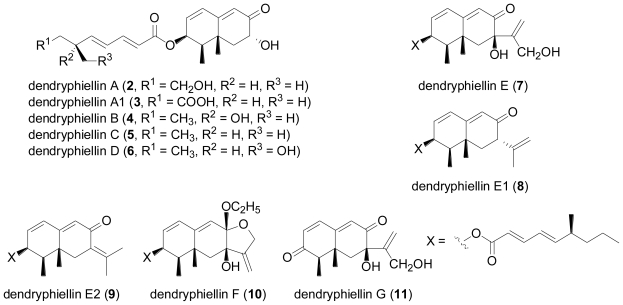


Subsequent studies by the same research group resulted in the report of further trinor-eremophilanes, dendryphiellins B (**4**), C (**5**), and D (**6**), together with the intact eremophilanes, dendryphiellins E (**7**), F (**10**), and G (**11**)[6]. Dendryphiellins E (**7**) and G (**11**) were found to exist in equilibrium with their corresponding hemiacetals, while the ethyl acetal in dendryphiellin F (**10**) probably formed during the isolation of dendryphiellin E (**7**) with ethanol. Additionally, the free carboxylic acids comprising the side chains of **2** and **4** were isolated from extracts of *D. salina*. The configuration of the methyl groups in the side chains was established by synthesis of the corresponding acids. Further investigation of the same deuteromycete yielded another trinor-eremophilane, dendryphiellin A1 (**3**), as well as the two intact eremophilanes, dendryphiellins E1 (**8**) and E2 (**9**)[7].

Two new eremophilane sesquiterpenes, 3-acetyl-9,7(11)-dien-7α-hydroxy-8-oxoeremophilane (**12**) and 3-acetyl-13-deoxyphomenone (**13**) were produced by the marine fungus *Penicillium* sp. BL27-2, isolated from sea mud in the Bering sea [8]. **13** had been synthesized in the course of the structure elucidation of sporogen A0 I from a mycophilic *Hansfordia* sp. [9], but had not been previously reported as a natural product. The epoxide **13** displayed pronounced cytotoxic activity in the nanomolar range when tested against three different cell lines, while the ring-opened alcohol **12** was less active by several orders of magnitude.

**Figure:**
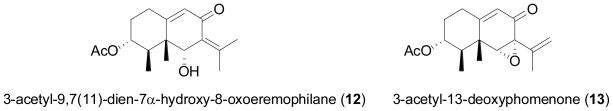


From the extract of the fungus *Microsphaeropsis* sp., isolated from the sponge *Myxilla incrustans*, the eremophilane metabolite microsphaeropsisin (**14**) was obtained [10]. Compound **14** is characterised by a *trans*-configuration of CH**3**-14 and CH**3**-15 which is only rarely observed for eremophilane sesquiterpenes, and displayed moderate antifungal activity towards *Eurotium repens* and *Ustilago violacea*.

**Figure:**
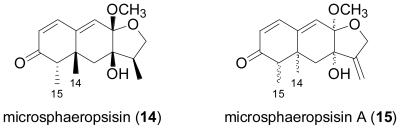


An undisclosed mangrove endophytic fungal strain yielded the closely related new sesquiterpene, microsphaeropsisin A (**15**)[11]. Unfortunately, due to its instability neither the relative stereochemistry nor the biological activity of **15** could be investigated.

**Figure:**
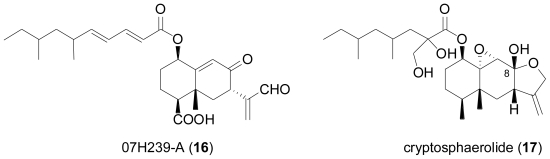


Fungal strain LL-07H239 was obtained from a frond of the mangrove palm, *Nypa* sp. and identified as belonging to the Xylariaceae based on its DNA sequence [12]. Upon fermentation in potato dextrose broth, the novel acylated eremophilane sesquiterpene 07H239-A (**16**) was detected, which displayed cytotoxicity toward a variety of cancer cell lines. Interestingly, the 3-oxoprop-1-en-2-yl-sustituted eremophilane carboxylic acid moiety in **16** is also present in integric acid, an HIV-1 integrase inhibitor produced by a terrestrial *Xylaria* sp. [13,14], while the branched unsaturated fatty acid substituent in **16** is identical to the one present in the structurally related sesquiterpenoid Sch 420789, which displayed phospholipase D inhibiting properties and was obtained from an unidentified fungus [15].

The marine-derived ascomycete fungal strain CNL-523 was isolated from an unidentified ascidian in the Bahamas, and based on its DNA sequence, it was found to belong to the family Diatrypaceae, and to be related to the genus *Cryptosphaeria* [16]. Chemical analysis resulted in the isolation of cryptosphaerolide (**17**), an ester-substituted sesquiterpenoid related to the eremophilane class, but structurally unusual in terms of the presence of an exomethylene function. The same sesquiterpenoid skeleton is present in the recently described berkleasmin A, a metabolite of the terrestrial saprobic fungus, *Berkleasmium nigroapicale* [17]. In the Mcl-1/Bak fluorescence resonance energy transfer (FRET) assay, **17** displayed inhibitory activity towards the Mcl-1 protein, a cancer drug target involved in apoptosis. In addition, **17** also showed significant cytotoxicity against the HCT-116 human colon carcinoma cell line, with IC_50_ values in the lower μM range. Its 8-*O*-methylated congener exhibited similar bioactivity, while the free alcohol resulting from cleavage of the ester substituent was neither cytotoxic nor active in the Mcl-1/Bak FRET assay.

Peribysins A–J (**18**–**27**) are a group of eremophilane-type sesquiterpenoids produced by *Periconia byssoides* which was isolated from the sea hare *Aplysia kurodai* [18–21]. They have raised considerable interest and have become the subject of synthetic efforts due to their ability of inhibit the adhesion of human-leukemia HL-60 cells to HUVEC at lower μM concentration, and thus are two orders of magnitude more potent than the standard control in this assay system, herbimycin A.

**Figure:**
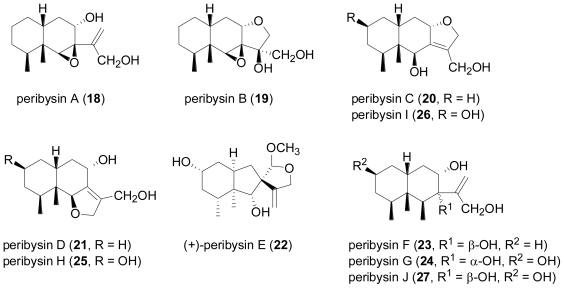


While the initial structure proposals for peribysins C (**20**) and D (**21**) suggested highly unusual furofuran skeletons, the structures were later revised based on CAST/CNMR prediction of ^13^C-NMR chemical shift values and geometric analyses with molecular and quantum mechanics calculations [22]. The absolute configuration for peribysin E (**22**) was initially established by the modified Mosher’s method, but later revised by total synthesis [23,24]. Interestingly, biological activity was only observed for the natural (+)-peribysin E, but not for its enantiomer.

A salt-water culture of an unidentified marine fungus, isolated from the marine sponge *Jaspis* aff. *johnstoni* yielded three new sesquiterpenes, chloriolins A (**28**), B (**29**), and C (**30**), chlorinated analogs of the terrestrial coriolin-type sesquiterpenes, besides the known coriolin B and dihydrocoriolin C [25]. Coriolins have been reported from the terrestrial basidiomycete, *Coriolus consors* [26–28]. Like the coriolins, **29** and **30** possess a hirsutane sesquiterpene skeleton, while **28** is probably derived via oxidative degradation, extruding C-5 and reverting the stereochemistry at C-3.

**Figure:**
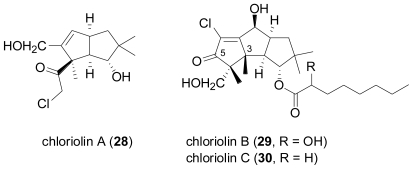


In a follow-up study by the same research group, the salt water culture of another unidentified fungus, this time obtained from the sponge *Haliclona* sp. was shown to produce several new hirsutane sesquiterpenes, hirsutanols A–C (**31**–**33**) and *ent*-gloeosteretriol (**35**)[29]. The latter compound is the enantiomer of the known gloeosteretriol from the terrestrial *Gloeostereum incarnatum* [30], and also shares the same planar structure with arthrosporol from a terrestrial arthroconidial fungus [31]. Under similar salt-water fermentation conditions, several terrestrial isolates of *Coriolus consors* were investigated, and one ATCC-derived culture provided hirsutanol D (**34**), featuring the new isohirsutane skeleton. However, none of the different fungi was able to produce chlorinated compounds. Hirsutanol A (**31**) and *ent*-gloeosteretriol (**35**) displayed antibiotic activity towards *B. subtilis*.

**Figure:**
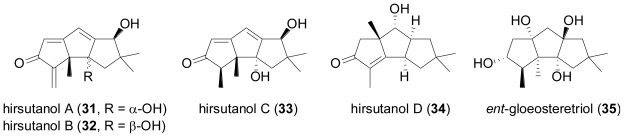


The marine fungus *Aspergillus versicolor*, isolated from the surface of the Caribbean green alga *Penicillus capitatus*, yielded four new sesquiterpenoid nitrobenzoyl esters (**36**–**39**), belonging to the cinnamolide class of drimane sesquiterpenes [32]. Compound **36** was responsible for essentially all of the HCT-116 colon carcinoma cell cytotoxicity observed for the crude extract, and displayed a mean LC_50_ of 1.1 μg mL^−1^ in the NCI’s 60 cell-line panel. In a parallel study conducted by a different research group, **36** was also isolated from a total of five strains of *Aspergillus insulicola* that were obtained from the green algae *Penicillus* sp. and *Batophora* sp., an unidentified green alga, and from a decaying leaf of the mangrove plant *Rhizophora mangle* from the Bahamas [33]. By these authors, **36** was given the name insulicolide A, and HPLC diode-array analyses revealed its presence in the extracts of several terrestrial isolates of *Aspergillus versicolor* as well as in some extracts of terrestrial isolates of *A. bridgeri* and some isolates of *A. sclerotiorum.*

**Figure:**
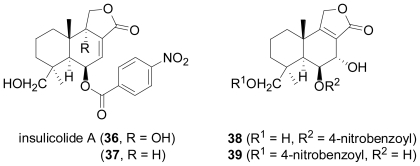


Seven new drimane sesquiterpenoids, hydroxylated derivatives of drim-7-en-6-one (**40**–**42**) and esters of 6β,9α-dihydroxy-5α-drim-7-en-11,12-olide with polyunsaturated acid substituents at C-6 (**45**–**48**), together with the known compounds deoxyuvidin B (**43**), strobilactone B (**44**), and RES-1149-2 (**49**), were obtained from cultures of the fungus *Aspergillus ustus*, which was isolated from the Mediterranean sponge *Suberites domuncula* [34,35].

**Figure:**
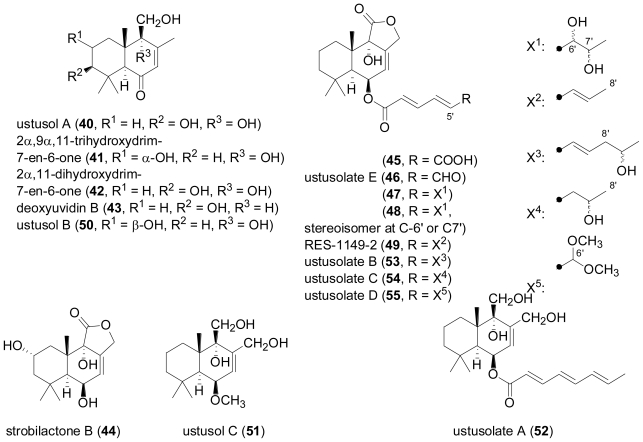


Compounds **45**, **46**, and **49** showed cytotoxic activity against a panel of tumor cell lines, and **46** was the most active with an EC_50_ value of 0.6 μg mL^−1^ against the L5178Y cell line. In a study which was published almost simultaneously, another isolate of *Aspergillus ustus*, obtained from the rhizosphere soil of the mangrove plant *Bruguiera gymnorrhiza* in Hainan, China, produced eight drimane sesquiterpenes, termed ustusols A–C (**40**, **50**, **51**) and ustusolates A–E (**52**–**55**, **46**), out of which two proved to be identical to the ones reported from the sponge-derived fungal strain mentioned above, besides another occurrence of RES-1149-2 (**49**)[36]. In this latter report, the absolute configuration of **40** was established based on its CD spectrum and the octant rule for cyclohexenones, and **46** and **54** were found to exhibit moderate cytotoxicity, while **52** was weakly active. It is noteworthy that compounds **41** and **50**, reported from the two research groups as C-2 epimers, displayed virtually identical ^13^C NMR data, and are thus very likely to be identical. RES-1149-2 (**49**) had previously been described as a metabolite from *A. ustus* var. *pseudoreflectus* isolated from a soil sample [37,38], and was found to act as endothelin type B receptor antagonist [39].

The fungus *Cadophora malorum*, isolated from the green alga *Enteromorpha* sp. was subjected to long-term fermentation in a medium supplemented with sea salt. Chemical analysis revealed the presence of the known (+)-sclerosporin (**56**), besides the four new hydroxylated derivatives, 15-hydroxysclerosporin (**57**), 12-hydroxysclerosporin (**58**), 11-hydroxysclerosporin (**59**), and 8-hydroxysclerosporin (**60**)[40]. Sclerosporin was initially characterised as a sporogenic metabolite of a terrestrial isolate of *Sclerotinia fruticula*, and is a rare example of a fungal-derived cadinane-type sesquiterpene [41,42]. The new compounds **57**–**60** were subjected to a variety of assays, but were found devoid of significant biological activity, apart from **60** which showed a weak fat-accumulation inhibitory activity against 3T3-L1 murine adipocytes.

**Figure:**
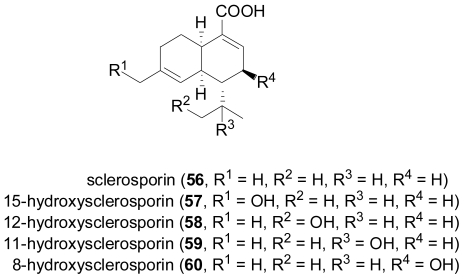


Four new phenolic bisabolane-type sesquiterpenoids, (+)-methyl sydowate (**61**), 7-deoxy-7,14-didehydrosydonic acid (**62**), 7-deoxy-7,8-didehydrosydonic acid (**63**), and (+)-sydowic acid (**64**), together with the known (+)-sydonic acid (**65**) were isolated from a marine-derived *Aspergillus* sp., which was in turn isolated from the gorgonian *Dichotella gemmacea* collected from the South China Sea [43]. The enatiomer of **64**, (−)-sydowic acid as well as the corresponding racemate, and sydonic acid (**65**) had previously been obtained from terrestrial strains of *Aspergillus sydowi* [44–46]. **61**, **64** and **65** exhibited weak antibacterial activity against *Staphylococcus aureus*, but were inactive against methicillin-resistant *S. aureus*.

The fungus *Verticillium tenerum*, isolated from an unidentified marine alga, yielded two new hydroxylated bisabolane-type sesquiterpenes, verticinol A (**66**) and B (**67**)[47]. Although the two new compounds were tested for a variety of effects, *i.e.*, antibacterial, antifungal, antialgal, antiplasmodial, antiviral, and cytotoxic activity as well as protein kinase inhibition or fat-accumulation inhibitory activity against 3T3-L1 murine adipocytes, they did not display significant activity in any of these test systems.

**Figure:**
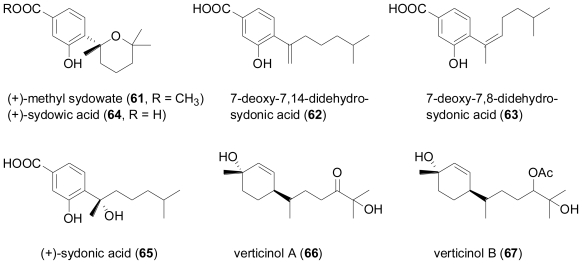


Two new bisabolane sesquiterpenoids, (+)-curcutetraol (**68**) and (−)-curcutriolamide (**69**) were obtained in a co-cultivation experiment of the marine bacterium CNH-741 and the fungus CNC-979, isolated from marine sediment [48]. The absolute configuration of **68** was determined by comparison of its experimental CD spectrum with the spectra predicted by quantum-chemical CD calculations. Although **68** and **69** bear structural similarity to known bisabolanes from terrestrial fungi such as sydonol from an unidentified member of the genus *Aspergillus* [49], or waraterpol from *Penicillium* sp. [50], it is not possible to definitely assign the actual producing organism in the co-cultivation study, or to decide whether the biosynthesis of **68** and **69** might have been induced by the presence of the other microorganism. An unidentified filamentous fungus, collected from driftwood in New Caledonia, was found to produce the bisabolane sesquiterpene, (−)-α-bisabolol (**70**)[51], which hitherto was only known as a typical plant metabolite, for example from chamomile (*Matricaria recutita*).

**Figure:**
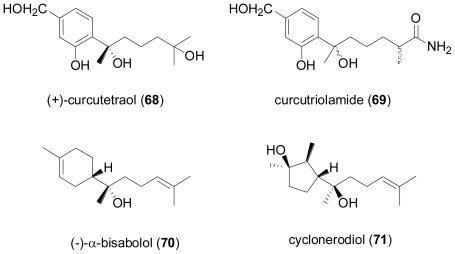


Cyclonerodiol (**71**) is a farnesane sequiterpenediol which was originally described from terrestrial plants, but later discovered to have a widespread occurrence in fungi, including the genera *Giberella*, *Fusarium*, *Trichoderma*, and *Trichothecium*. Occasionally, **71** has also been detected in marine-derived fungi; examples include fungi of the genera *Botrytis* [52] and *Myrothecium* [53] as well as an unidentified fungus [54], obtained from the green alga *Enteromorpha compressa* and the red alga *Gracillaria verrucosa*, respectively.

The fungus *Drechslera dematioidea* was isolated from the inner tissue of the marine red alga *Liagora viscida*, and proved to be a very rich source of new sesquiterpene derivatives [55]. Fermentation yielded ten new sesquiterpenoids, namely isosativenetriol (**72**), drechslerines A–G (**73**–**79**), 9-hydroxyhelminthosporol (**80**), and sativene epoxide (**81**). In addition, a series of known sesquiterpenes were also detected, including helminthosporol, originally described as a plant growth regulator from *Helminthosporium sativum* [56], *cis*-sativenediol, a plant growth promotor produced by the same fungus and also by *Cochliobolus setariae* [57], and (+)-secolongifolene diol, again produced by *Helminthosporium sativum* and also by *H. victoriae* [58]. From a structural point of view, the sesquiterpenes produced by *Drechslera dematioidea* belong to four different classes of irregular terpenes, *i.e.*, isosativene (**72**), seco-sativene or helminthosporene (helminthosporol and **73**–**80**), sativene (*cis*-sativenediol and **81**), and secolongifolene (secolongifolene diol). Most of the compounds displayed modest antifungal activity and were devoid of antialgal activity towards the green alga *Chlorella fusca*, and were also inactive against brine shrimp, nematodes, and *Mycobacterium tuberculosis*. However, drechslerins E (**77**) and G (**79**) and helminthosporol exhibited antimalarial activity against *Plasmodium falciparum*.

**Figure:**
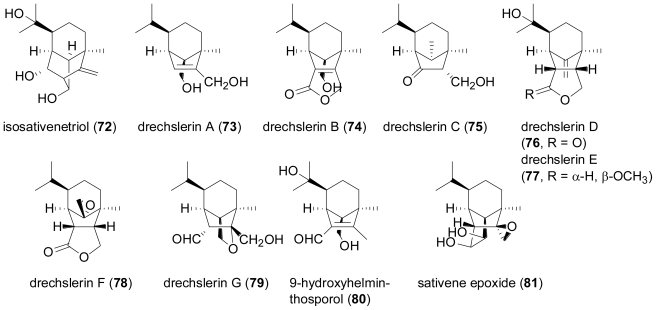


A culture of the marine fungus *Kallichroma tethys* grown in an enriched seawater medium gave the new tricyclic sesquiterpene, isoculmorin (**82**), besides traces of its isomer, culmorin (**83**)[59].

**Figure:**
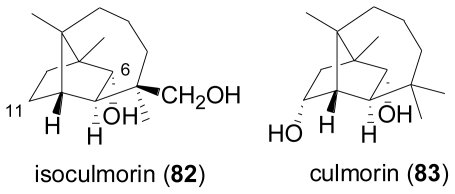


Compound **82** differs from culmorin and all other known hydroxyculmorin derivatives in the lack of oxygenation at C-11. Culmorin and its hydroxylated congeners, but not isoculmorin, are widely distributed in various terrestrial *Fusarium* species, commonly found as pathogens on cereal crops [60,61]. Very often they co-occur with thrichotecenes such as deoxynivalenol (DON), which are also sesquiterpenes, but are characterised by a heavily rearranged and oxygenated carbon framework. Very recently, a terpene cyclase termed CLM1 has been identified in *Fusarium graminearum*, which produces longiborneol (11-deoxyculmorin) and is required for culmorin biosynthesis [62].

Trichothecenes are well-known sesquiterpene toxins produced by several fungi from the genera *Fusarium*, *Myrothecium*, *Trichothecium*, and *Trichoderma*, most of which are parasitic on cereals such as maize, wheat, rye, barley, and rice [63]. Very often, besides a highly rearranged sesquiterpenoid-derived part, trichothecenes carry two additional polyketide-derived hydroxyacid moieties at C-4 and C-15, which can be linked *via* an ether or an ester bridge to give rise to a macrocycle.

From the marine environment, the first trichothecene producing fungi, *Acremonium neo-caledoniae* from New Caledonia [64], and *Myrothecium roridum* from Palau [65], both originated from wood samples. The deuteromycete, *Acremonium neo-caledoniae* was identified based on morphological criteria and by 18S rDNA sequencing. The considerable cyotoxicity of its crude extract could be attributed to the presence of the known trichothecenes verrucarin A, isororidin A, and the new congener verrol 4-acetate (**93**)[64]. From *Myrothecium roridum*, a new member of the roridin family, 12,13-deoxyroridin E (**91**) was obtained, which differs from the known roridin E (**86**) by the lack of the epoxide function in the sesquiterpenoid part. Although **91** displayed IC_50_ values of 25 and 15 ng mL^−1^ towards HL-60 and L1210 cell lines, respectively, its activity was reduced about 80 fold in comparison to **86** [65]. In a subsequent study, the same fungal strain was reported to produce three new macrocyclic trichothecenes, 12′-hydroxyroridin E (**87**), roridin Q (**88**), and 2′,3′-deoxyroritoxin D (**98**), while one new compound, roridin R (2′,3′-dihydro-2′-hydroxyroridin H, **97**), was isolated from *Myrothecium* sp., together with the known foridins A and H, and isororidin E [66]. **88** is characterized by a unique ether moiety at C-13′ and thus contains a third hydroxyacid moiety. **87**, **88** and **97** displayed cytotoxic activity towards the murine leukemia cell line L1210 with IC_50_ values of 0.19, 31.2, and 0.45 μM, respectively, while **98** showed antifungal activity against *S. cerevisiae* at 1 μg/disc. A saltwater culture of *Myrothecium verrucaria*, isolated from the Hawaiian sponge *Spongia* sp. yielded three new trichothecenes, 3-hydroxyroridin E (**89**), 13′-acetyltrichoverrin B (**94**), and miophytocen C (**95**), together with the known roridin A (**84**), roridin L (**90**), verrucarin M (**92**), roridin M (**96**), verrucarin A, isororidin A, epiroridin E, trichoverrin A, and trichoverrin [67]. When establishing the stereostructures of the new compounds by a combination of NMR spectroscopy and chemical transformations, the previously unreported stereochemistry of **90**, **92**, and **96** were also elucidated. All isolated compounds were shown to possess significant cytotoxicity against murine and human tumor cell lines, apart from **95** which lacks the epoxide function present in all other congeners. A total of 16 fungal strains were isolated from various tissues of the fish, *Argyrosomus argentatus* (white croaker). Screening for antifungal activity against the human pathogenic *C. albicans*, *Aspergillus niger*, and *Trichophyton rubrum* identified a *Myrothecium* sp. as the most active isolate, which produced the known trichothecenes roridin A (**84**), verrucarin A, and 8β-acetoxyroridin H [68]. In a systematic screening for antimicrobial activity towards gram-positive and gram-negative bacteria as well as *S. cerevisiae* and the two plant pathogenic fungi *Sclerotinia sclerotiorum* and *Magnaporthe grisea*, a total of nine fungal strains obtained from the sponge *Axinella* sp. collected in the South China Sea were investigated. The most active isolate was identified as *Myrothecium* sp., and chemical analysis revealed the presence of the known roridin A (**84**) and roridin D (**85**), which were found to be responsible for the antifungal activity [69].

**Figure:**
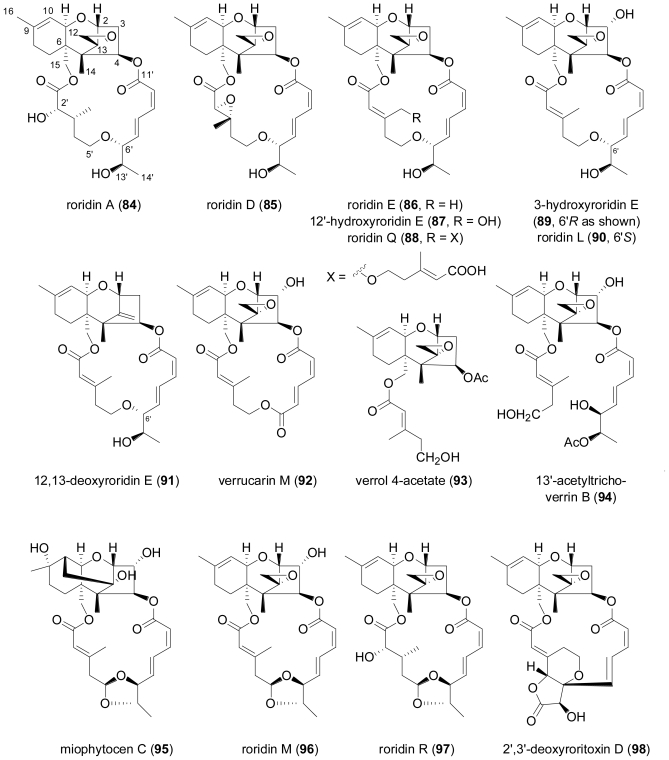


### 2.3. Diterpenes

Perhaps the most intriguing class of fungal diterpenes are the phomactins, a group of platelet-activating factor (PAF) antagonists produced by the fungus Phoma sp. obtained from the shell of the crab Chionoecetes opilio.

**Figure:**
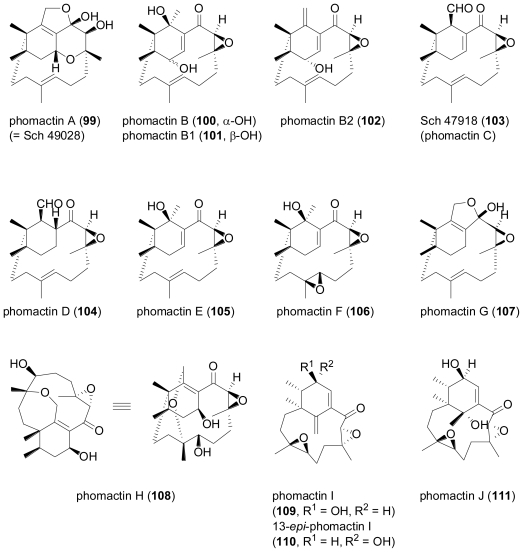


After the initial report of phomactin A (**99**)[70], further derivatives phomactins B (**100**), B1 (**101**), B2 (**102**), C (**103**), D (**104**)[71], and subsequently phomactins E (**105**), F (**106**) and G (**107**)[72] were reported from the same fungal strain. Of this series, phomactin D (**104**) proved the most potent congener and inhibited PAF receptor binding with an IC_50_ value of 0.12 μM, and platelet aggregation with an IC_50_ value of 0.80 μM. Independently, researchers from Scheringh Plough in the US reported further phomactin derivatives Sch 47918 (**103**, identical to phomactin C) as well as Sch 49026, Sch 49027 and Sch 49028 (later found to correspond to phomactin A, **99**) from a terrestrial *Phoma* sp., isolated from a leaf litter sample of mixed *Quercus* species, which was collected in a second growth mixed hardwood lot in Baton Rouge, Louisiana [73,74]. Later on, an unidentified marine-derived fungus that was isolated from the surface of the marine brown alga *Ishige okamurae*, and based on DNA sequence analysis was grouped into the order Dothideales and thus was taxonomically not closely related to the genus *Phoma*, was identified as the producer of phomactin H (**108**)[75], and most recently, three further derivatives, phomactin I (**109**), 13-*epi*-phomactin I (**110**), and phomactin J (**111**)[76]. Due to their interesting biological properties, phomactins have been the subject of numerous synthetic efforts, including successful total syntheses and the preparation of numerous synthetic derivatives which greatly contributed to a better understanding of structure-activity relationships, which has been reviewed in detail [77]. Interestingly, there is increasing evidence that phomactins share a common biogenetic origin with the plant-derived diterpene alkaloid paclitaxel (also known as taxol), with the precursor, geranylgeranyldiphophate, undergoing cyclisation to the common biosynthetic intermediate, verticillenyl cation, which then is further modified either into taxadiene, the precursor of paclitaxel, or alternatively, into phomactatriene, which would then give rise to the phomactins [78–80]. In a fascinating study, the induction of biosynthesis of four new pimarane diterpenoids, libertellenones A–D (**112**–**115**) was observed when of a marine α-proteobacterium (strain CNJ-328) was added to an established 3-day-old culture of the marine-derived fungus *Libertella* sp., isolated from an ascidian that was collected in the Bahamas [81].

**Figure:**
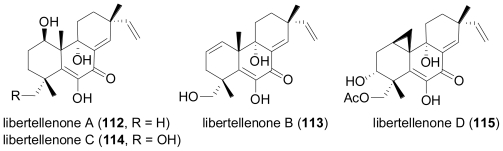


When cultured alone, neither the fungal strain nor the bacterium produced diterpenoid metabolites. Based on structural properties, libertellenones A–D (**112**–**115**) are very likely produced by the fungus, since pimarane diterpenes have never been reported from bacteria. Curiously, the marine bacterium CNJ-328, which is capable of inducing diterpenoid biosynthesis, is the same strain that induced the production of the chlorinated benzophenone antibiotic, pestalone in a culture of the fungus *Pestalotia* sp., as previously reported by the same research group [82]. However, libertellenones A–D (**112**–**115**) did not show antibiotic activity towards the inducing marine bacterium, nor were active against multidrug-resistant human pathogenic bacterial strains. When tested against the HCT-116 human adenocarcinoma cell line, libertellenone D (**115**) showed pronounced cytotoxic activity with an IC_50_ value of 0.76 μM, while **112**–**114** were less active by almost two orders of magnitude.

Chemical analysis of the endophytic fungus *Apiospora montagnei*, isolated from the alga *Polysiphonia violacea* led to the discovery of the new pimarane diterpene myrocin A (**116**)[83]. **116** is closely related to myrocin B, a diterpene previously isolated from the terrestrial fungus *Myrothecium verrucaria* [84].

**Figure:**
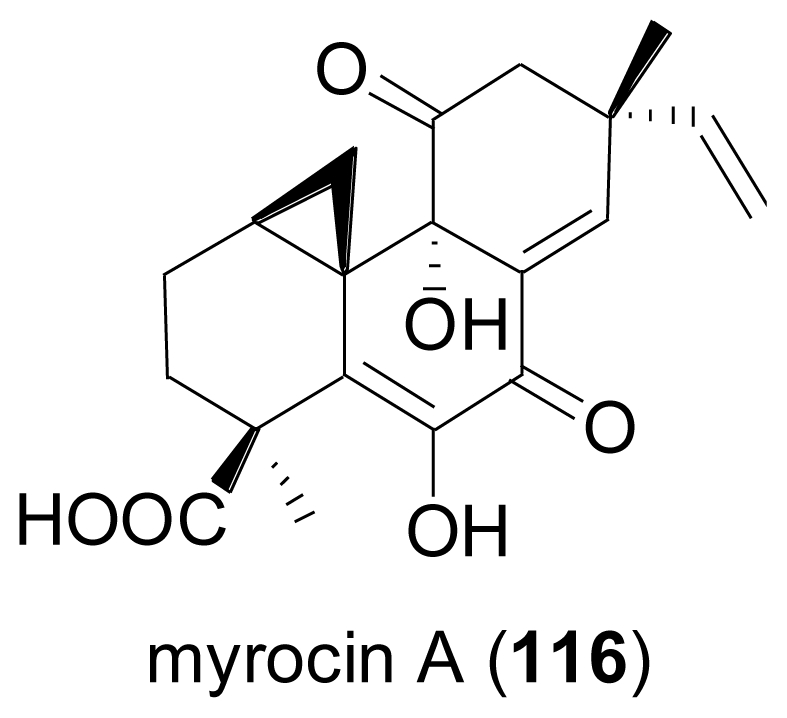


The fungus *Acremonium striatisporum* was isolated from superficial mycobiota of the sea cucumber, *Eupentacta fraudatrix* collected from the Sea of Japan. Over the course of eight years, it was repeatedly studied and proved to be an exceptionally rich source of new isopimaradiene diterpene glycosides, virescenosides N–X (**118**, **120**, **122**–**131**)[85–88].

**Figure:**
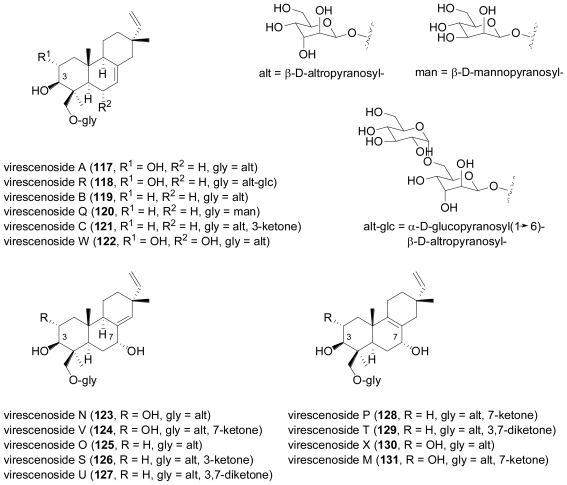


Additionally, the known virescenosides A (**117**), B (**119**), and C (**121**), previously described from terrestrial strains of *Oospora virescens* (later called *Acremonium luzulae*, while the currently accepted name is *Gliomastix luzulae*) were also obtained. In most virescenosides, the sugar is an unusual β-d-altropyranose. Most of the virescenosides showed cytotoxic effects on developing eggs of the sea urchin *Strongylocentrotus intermedius*, accompanied by cytotoxic activity against Ehrlich carcinoma cells. Hypoxysordarin (**132**) was isolated from the fermentation broth of the facultative marine *Hypoxylon croceum*, obtained from a mangrove estuary driftwood sample [89].

**Figure:**
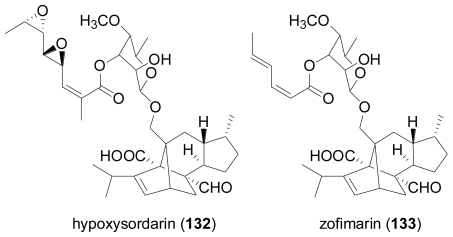


Compound **132** showed lower MICs against several filamentous fungi than its parent compound, sordarin which lacks the unsaturated short-chain fatty acid substituent at the sordarose sugar unit, and which was originally reported from the terrestrial fungus, *Sordaria araneosa* [90].

Structurally closely related is zofimarin (**133**), a compound which was isolated from the fungus *Zopfiella marina* obtained from a marine mud sample by Japanese researchers at Sankyo laboratories, and which only had been reported in the patent literature [91]. Sordarin-type diterpene glycosides are of great interest due to their ability to selectively inhibit fungal protein synthesis through their interaction with the elongation factor 2 (EF2), and show a widespread distribution among terrestrial, but only occasionally among marine-derived fungi [92].

### 2.4. Sesterterpenes

A *Fusarium* sp., tentatively identified as *F. heterosporum*, was isolated from driftwood in a mangrove habitat in the Bahamas. Fermentation in a seawater-based medium led to the discovery of two groups of sesterterpenes, neomangicols A–C (**134**–**136**)[93] and mangicols A–G (**137**–**143**)[94]. Very likely, neomangicol C (**136**) is an artefact resulting from conversion of neomangicol A (**134**) or B (**135**) by a complex dehydrohalogenation process.

**Figure:**
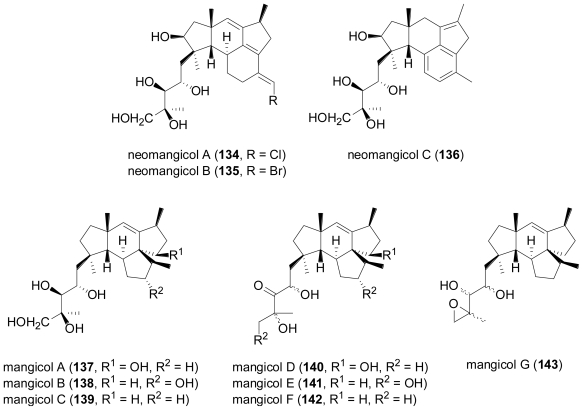


Mangicols A–G (**137**–**143**) possess an unprecedented spirotricyclic sesterterpene skeleton. Based on ^13^C-acetate labelling studies, a biosynthetic route starting from geranylfarnesyldisphosphate was proposed, which upon cyclisation would undergo various 1,2-alkyl shifts and 1,2-hydride shifts. A rearrangement of the proposed cationic intermediate would also account for the biosynthesis of the neomangicols via an analogous pathway. Neomangicols A (**134**) and B (**135**) and mangicols A–G (**137**–**143**) displayed moderate cytotoxic activity against a panel of cell lines, and additionally, neomangicol B (**135**) was active against *B. subtilis*, while mangicols A (**137**) and C (**139**) significantly inhibited phorbol myristate acetate-induced edema.

The ophiobolins represent a class of unusual sesterterpenes so far described for a number of terrestrial fungi, including representatives of the genera *Ophiobolus*, *Cochliobolus*, *Helminthosporium*, *Cephalosporium*, *Aspergillus*, and *Drechslera* [95]. From a culture of the fungus *Emericella variecolor* obtained from a marine sediment, two new congeners, 6-*epi*-ophiobolin G (**145**) and 6-*epi*-ophiobolin N (**146**), and six known ophiobolins were isolated [96].

**Figure:**
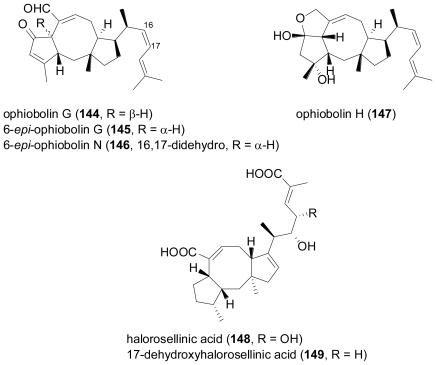


In the course of the structure elucidation, the configuration of the C-6 proton in ophiobolin G (**144**) was revised from α to β, and the previously not reported stereochemistry of ophiobolin H (**147**) was determined by spectroscopical means and chemical correlation with ophiobolin K. All of the isolated compounds showed cytotoxicity against a neuroblastoma cell line. When testing different culture media, it was found that cultivation of the fungus on solid media increased the yield in ophiobolins about ten-fold in comparison to fermentation in liquid media. In a subsequent report, ophiobolin H (**147**) was also detected in the culture broth of the fungus *Aspergillus ustus*, which was isolated from the Mediterranean sponge *Suberites domuncula* [35].

The obligate marine fungus *Halorosellinia oceanica*, collected in Thailand, was found to produce halorosellinic acid (**148**), an ophiobolane sesterterpene with weak antimalarial activity (MIC value of 200 μg mL^−1^)[97]. Re-examination of the same fungus provided another new ophiobolane sesterterpene, 17-dehydroxyhalorosellinic acid (**149**)[98].

A marine-derived *Aspergillus* sp. was found to produce a new sesterterpene epoxide-diol named aspergilloxide A (**150**)[99]. The carbon skeleton of **150**, for which the name asperane is proposed, represents a new addition to the architectural diversity of the sesterterpenoid class of secondary metabolites. When tested for cytotoxic properties, the acetylation product of **150** was moderately active against a human colon carcinoma cell line, while the parent compound was inactive.

**Figure:**
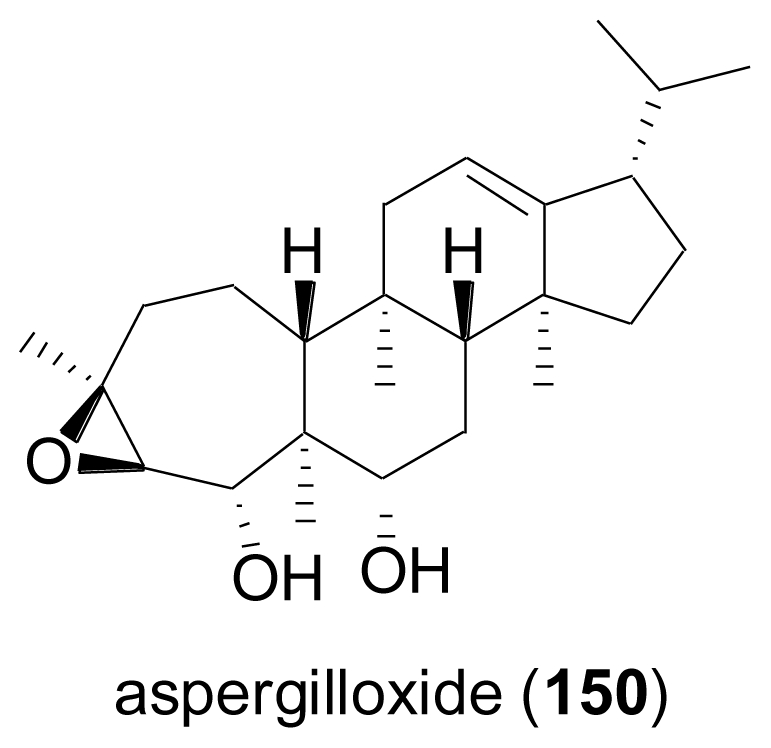


### 2.5. Triterpenes

The fungus *Phomopsis* sp. was isolated from the Chinese mangrove plant, *Hibiscus tiliaceus*. Chemical analysis revealed four new unusual ring A-*seco*-oleanes, namely 3,4-*seco*-olean-11,13-dien- 4,15α,22β,24-tetraol-3-oic acid (**151**), 3,4-*seco*-olean-11,13-dien-4,7β,22β,24-tetraol-3-oic acid (**152**), 3,4-*seco*-olean-13-en-4,7α,15α,22α,24-pentaol-3-oic acid (**153**), and 3,4-*seco*-olean-13-en- 4,15α,22α,24-tetraol-3-oic acid (**154**)[100].

**Figure:**
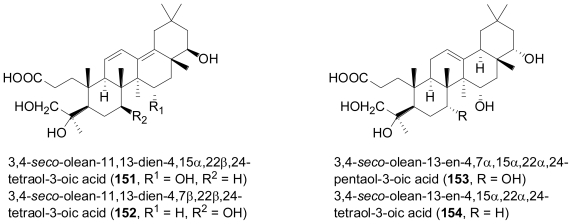


Oleane-type triterpenes are frequently found in terrestrial plants, but have only rarely been reported from microbial including fungal sources, while this investigation detected A-*seco*-oleanes for the first time in fungi. Interestingly, some fungi are able to converting oleananes into A-*seco*-oleanes, which might be relevant with regard to the biosynthetic origin of the compounds under study [101,102]. Besides a series of different metabolites from other biogenetic groups, the rearranged triterpene, 6β,16β-diacetoxy-25-hydroxy-3,7-dioxy-29-nordammara-1,17(20)-dien-21-oic acid (**155**) was isolated from a culture of the fungus *Aspergillus sydowi* PFW1, obtained from a driftwood sample collected from the beach of the island of Hainan in China [103]. Compound **155** displayed significant antibiotic activity towards *Escherichia coli*, *Bacillus subtilis* and *Micrococcus lysoleikticus*. The new friedelan triterpene, 3β-hydroxyfriedelan-17β-carboxylic acid (**156**) was isolated from an unidentified mangrove endophytic fungus [104].

Bioassay-guided fractionation following hemolytic properties led to isolation of the pentacyclic triterpenoid sapogenin miliacin (3β-methoxyolean-18-ene, **157**) from a culture of the fungus *Chaetomium olivaceum*, obtained from marine bottom sediments of the Kuriles islands [105]. **157** had initially been described from the seeds of switch grass, *Panicum miliaceum* [106] and later found to occur rather widespread in higher terrestrial plants, but had not been described as a metabolite of marine-derived fungi before.

**Figure:**
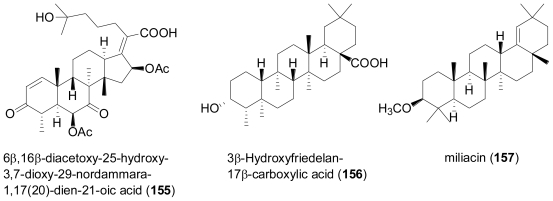


### 2.6. Steroids

The fungus *Gymnascella dankaliensis* was isolated from the Japanese sponge *Halichondria japonica*, and yielded a series of structurally unusual steroid-type compounds, the pattern of which varied depending on media composition [107,108]. Dankasterones A (**158**) and B (**159**) were obtained when glucose in the original medium was replaced by soluble starch, while gymnasterones A (**160**), B (**161**), C (**162**) and D (**163**) were isolated from malt-glucose-yeast media. **158** and **159** are most unusual steroids possessing a 13(14→8)*abeo*-8-ergostane skeleton, which so far only has been described once from nature, resulting from a photochemical reaction of the insect molting hormone, 20R-hydroxyecdysone [109].

**Figure:**
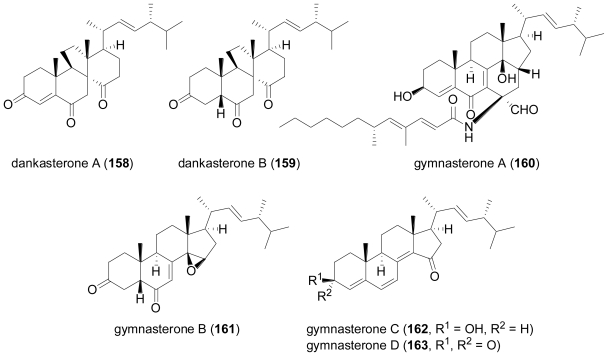


On the other hand, **160** is structurally intriguing since it represents an unprecedented steroid alkaloid with an additional ring and an amide-linked side chain derived from gymnastatins, a group of polyketides likewise described from *Gymnascella dankaliensis*. **161** is a rare example of steroids with an epoxide-substituted D ring, while **162** and **163** contain an unusual 4,6,8(14)-conjugated triene system. **158**, **159** and **161**–**163** exhibited significant growth inhibition against the murine P388 cancer cell line, whereas **158** also exhibited potent growth inhibition against human cancer cell lines.

**Figure:**
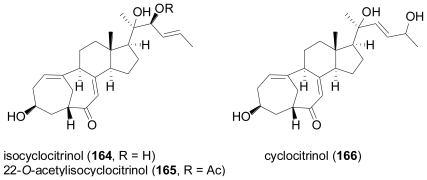


Isocyclocitrinol (**164**) and its 22-acetyl derivative (**165**) were detected in the culture broth of a *Penicillium citrinum* which had been isolated from a sponge of the genus *Axinella* collected in Papua New Guinea [110]. **164** and **165** feature a most unusual bicyclo[4.4.1] A/B ring steroid system which arises from incorporation of 19-CH_3_ into the ring by 1,2-migration, yielding two fused seven-membered rings with a double bond at the bridgehead. The novel carbon framework of **165** was confirmed by X-ray crystallographic analysis. Structurally, both isocyclocitrinol congeners are related to cyclocitrinol (**166**) isolated from a terrestrial *Penicillium citrinum* [111], the structure of which was revised in the course of the structure elucidation of isocyclocitrinol. **164** and **165** showed weak antibacterial activity against *Staphylococcus epidermidis* and *Enterococcus durans*.

The marine-derived fungus *Rhizopus* sp., isolated from the bryozoan *Bugula* sp. collected in Jiaozhou Bay, China, yielded six new ergosterols, 3β-hydroxy-(22*E*,24*R*)-ergosta-5,8,22-trien-7,15-dione (**167**), 3β-hydroxy-(22*E*,24*R*)-ergosta-5,8,14,22-tetraen-7-one (**168**), 3β,15β-dihydroxy-(22*E*,24*R*)-ergosta- 5,8(14),22-trien-7-one (**169**), 3β,15β-dihydroxy-(22*E*,24*R*)-ergosta-5,8(14),22-trien-7-one (**170**), 3β-hydroxyl-(22*E*,24*R*)-ergosta-5,8(14),22-trien-7,15-dione (**171**), and 5α,8α-epidioxy-23,24(*R*)- dimethylcholesta-6,9(11),22-trien-3β-ol (**172**)[112]. All compounds showed cytotoxic activity to varying degrees against four different cancer cell lines.

**Figure:**
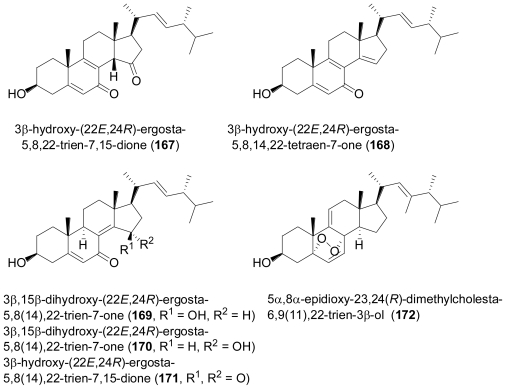


Ergosterimide (**173**) is an unusual steroid derivative, formally a Diels-Alder adduct of maleimide and (22*E*,24*R*)-ergosta-5,7,14-trien-3β-ol. The compound was obtained when investigating the endophytic fungus *Aspergillus niger* EN-13, cultured from the Chinese marine brown alga, *Colpomenia sinuosa* [113]. Maleimide is widely used for technical applications, and due to its high reactivity is commonly used as an educt for Diels-Alder reactions in synthetic laboratories. If maleimide indeed was a metabolite of the fungus under study, **173** would represent the first natural Diels-Alder adduct of this type.

**Figure:**
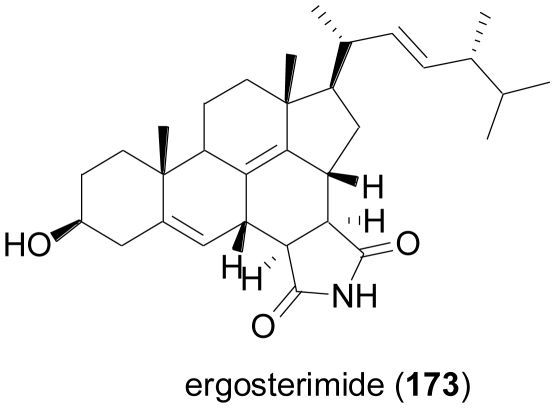


Six new fatty acid esters of the known steroids (22*E*)-ergosta-7,22-diene-3β,5α,6α-triol (**174**–**177**) and (22*E*)-ergosta-7,22-diene-3β,5α,6β-triol (**178**, **179**) were isolated from the fungus *Aspergillus awamori* isolated from soil around the mangrove plant *Acrostichum speciosum* in Hainan, China, besides various known steroids and their esters [114]. All compounds exhibited mild cytotoxic activity against B16 and SMMC-7721 cell lines. Further analysis yielded the two new oxidized sterols, (22*E*)-7α-methoxy-5α,6αepoxyergosta-8(14),22-dien-3β-ol (**180**) and (22*E*)-3β-hydroxy- 5α,6α,8α,14α-diepoxyergosta-22-en-7-one (**181**) which were mildly cytotoxic towards the lung cancer cell line A549 [115]. Conformational analysis on the basis of the observed NOEs in the ROESY spectrum indicated that the cyclohexene oxide system in ring B of **180** adopted an *endo*-boat rather than a half-chair conformation.

**Figure:**
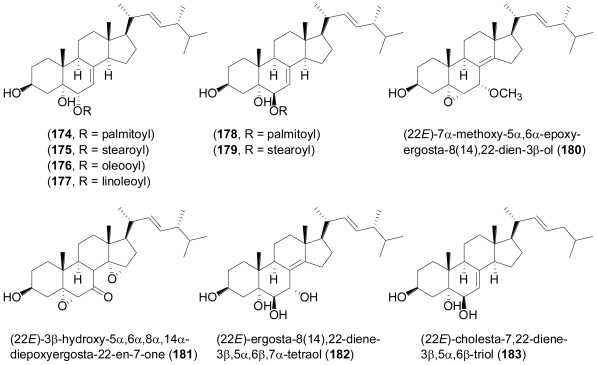


The fungus *Penicillium* sp. was obtained from an undisclosed moss collected from the South Pole. Chemical analysis revealed the presence of a new sterol, ergosta-8(14),22-diene-3β,5α,6β,7α-tetraol (**182**), together with four known sterols. **182** exhibited pronounced cytotoxicity against the human liver cancer cell line Hep G with an IC_50_ value of 10.4 μg mL^−1^ [116].

The known cholesta-7,22-diene-3β,5α,6β-triol (**183**) was identified from a marine *Trichoderma* sp. that was isolated from deep sea sediment of the South China Sea [117]. When tested for biological activity, it displayed weak cytotoxicity towards A549, inhibited Taq DNA polymerase with an IC_50_ value of 0.45 mM, and also exhibited moderately inhibitory activity against HIV-1 protease, but was devoid of antimicrobial activity.

### 2.7. Tetraterpenes (Carotenoids)

The occurrence of tetraterpenes or carotenoids has only rarely been studied for marine-derived fungi. One report describes the first neurosporaxanthin glycoside, neurosporaxanthin β-d-glucopyranoside (**184**), as a metabolite of a *Fusarium* sp. isolated from the seawater surface in Japan [118]. In addition, the known carotenoids neurosporaxanthin, β-carotene, γ-carotene, and torulene were also identified. Since these pigments have strong ^1^O_2_ quenching activities, the authors speculated that they might contribute to the survival of the fungus in a harsh environment, exposed to active oxygen species and free radicals generated by intense irradiation with strong sunlight.

**Figure:**
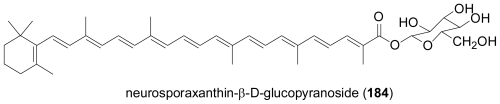


## 3. Conclusions

Terpenes from marine-derived fungi show a pronounced degree of structural diversity, and many of them have aroused interest from synthetic chemists and the pharmaceutical industry alike, due to their interesting biological and pharmacological properties. Examples include the phomactins and the peribysins as “originally marine:, and the trichothecenes and the sordarins as “originally terrestrial” groups of compounds, even though it is becoming increasingly evident that these distinctions are more or less coincidental, with further isolation studies yielding examples of producing organisms from a wider range of ecosystems. Thus, even if there are no exclusive classes of marine fungal terpenes, marine fungi represent an extremely valuable addition to nature’s vast repository of unique structures with intriguing activities.
